# A novel mitochondrial function-associated programmed cell death-related prognostic signature for predicting the prognosis of early breast cancer

**DOI:** 10.3389/fgene.2024.1406426

**Published:** 2024-07-02

**Authors:** Jian Wang, Haiming Jiang

**Affiliations:** ^1^ Department of Breast Vascular Intervention, Qingzhou People’s Hospital, Qingzhou, Shandong, China; ^2^ Department of General Surgery, Qingzhou People’s Hospital, Qingzhou, Shandong, China

**Keywords:** breast cancer, mitochondrial function, programmed cell death, immune infiltration, prognostic model, nomogram

## Abstract

**Purpose:** To screen mitochondrial function-associated PCD-related biomarkers and construct a risk model for predicting the prognosis of early breast cancer.

**Methods:** Data on gene expression levels and clinical information were obtained from the TCGA database, and GSE42568 and GSE58812 datasets were obtained from GEO database. The mitochondrial function-associated programmed cell death (PCD) related genes in early breast cancer were identified, then LASSO logistic regression, SVM-RFE, random forest (RF), and multiple Cox logistic regression analysis were employed to construct a prognostic risk model. Differences in immune infiltration, drug sensitivity, and immunotherapy response were evaluated between groups. Lastly, the qRT-PCR was employed to confirm the key genes.

**Results:** Total 1,478 DEGs were screened between normal and early breast cancer groups, and these DEGs were involved in PI3K-Akt signaling pathway, focal adhesion, and ECM-receptor interaction pathways. Then total 178 mitochondrial function-associated PCD related genes were obtained, followed by a four mitochondrial function-associated PCD related genes prognostic model and nomogram were built. In addition, total 2 immune checkpoint genes were lowly expressed in the high-risk group, including CD47 and LAG3, and the fraction of some immune cells in high- and low-risk groups had significant difference, such as macrophage, eosinophil, mast cell, etc., and the Top3 chemotherapeutics with significant differences were included FH535, MK.2206, and bicalutamide. Finally, the qRT-qPCR results shown that the CREB3L1, CAPG, SPINT1 and GRK3 mRNA expression were in line with the bioinformatics analysis results.

**Conclusion:** Four mitochondrial function-associated PCD-related genes were identified, including CREB3L1, CAPG, SPINT1, and GRK3, and the prognostic risk model and nomogram were established for predicting the survival of early breast cancer patient. The chemotherapeutics, containing FH535, MK.2206, and bicalutamide, might be used for early breast cancer.

## Highlights

1. CREB3L1, CAPG, SPINT1, and GRK3 might be suitable for clinical application in early breast cancer treatment.
2. The 4 mitochondrial function**-**associated PCD-associated genes could be used as prognostic markers of early breast cancer.
3. The prognostic nomogram could accurately predict survival of early breast cancer.

## 1 Introduction

Malignant tumors are one of the major chronic diseases that seriously threaten the health of global people. Since the 21st century, the overall incidence and mortality of female breast cancer have shown an upward trend (Nolan et al., 2023; Roy et al., 2023). In 2020, the incidence and mortality of female breast cancer ranks among the cancers with the highest global incidence, with approximately 12.5% and 6.92% of the total incidence and mortality of malignant tumors, respectively (Sung et al., 2021). In China, whether in urban or rural areas, the incidence and mortality of breast cancer ranks first and four among female cancers, respectively, which has surpasses lung cancer in terms of incidence (Qiu et al., 2021). With the acceleration of the aging trend and the change of lifestyle, the incidence and death toll of breast cancer in Chinese women are expected to continue to rise, and will increase by 36.27% and 54.01% respectively by 2030 (Lei et al., 2021). Current treatment methods for breast cancer include surgical treatment, chemotherapy, radiotherapy, etc., but the survival rate of patients is still relatively low. Therefore, it is necessary to identify new biomarkers and develop effective prognostic predictors for patients with early breast cancer.

Mitochondria is a highly dynamic structural organelle, and its structure and proteins have high cellular phenotypic differences (Guo et al., 2023; Nguyen et al., 2023). Mitochondria play an important role in many aspects such as growth and development, metabolism, diseases, death, and biological evolution (Chen et al., 2023). Mitochondrial dysfunction can cause a range of diseases, such as metabolic disorders, cardiomyopathy, neurodegenerative diseases, and cancer (Calvo and Mootha, 2010; Nunnari and Suomalainen, 2012). Moreover, mitochondria play a critical role in providing energy for cellular functions, regulating cellular signaling pathways, and controlling programmed cell death (PCD) (Galluzzi et al., 2008). Mitochondria are the convergence point of multiple cell death induction pathways, which trigger various mechanisms of apoptotic and nonapoptotic PCD (Kamradt and Makarewich, 2023). It has been demonstrated that mitochondrial dysfunction and PCD mechanisms are crucial for the development and spread of malignant tumors (Kopecka et al., 2020; Saha et al., 2022; Nguyen et al., 2023). Nevertheless, the interaction between mitochondrial dysfunction and PCD in early breast cancer is still not fully understood, and the detailed functional studies of these processes in early breast cancer is also very limited.

**FIGURE 1:**
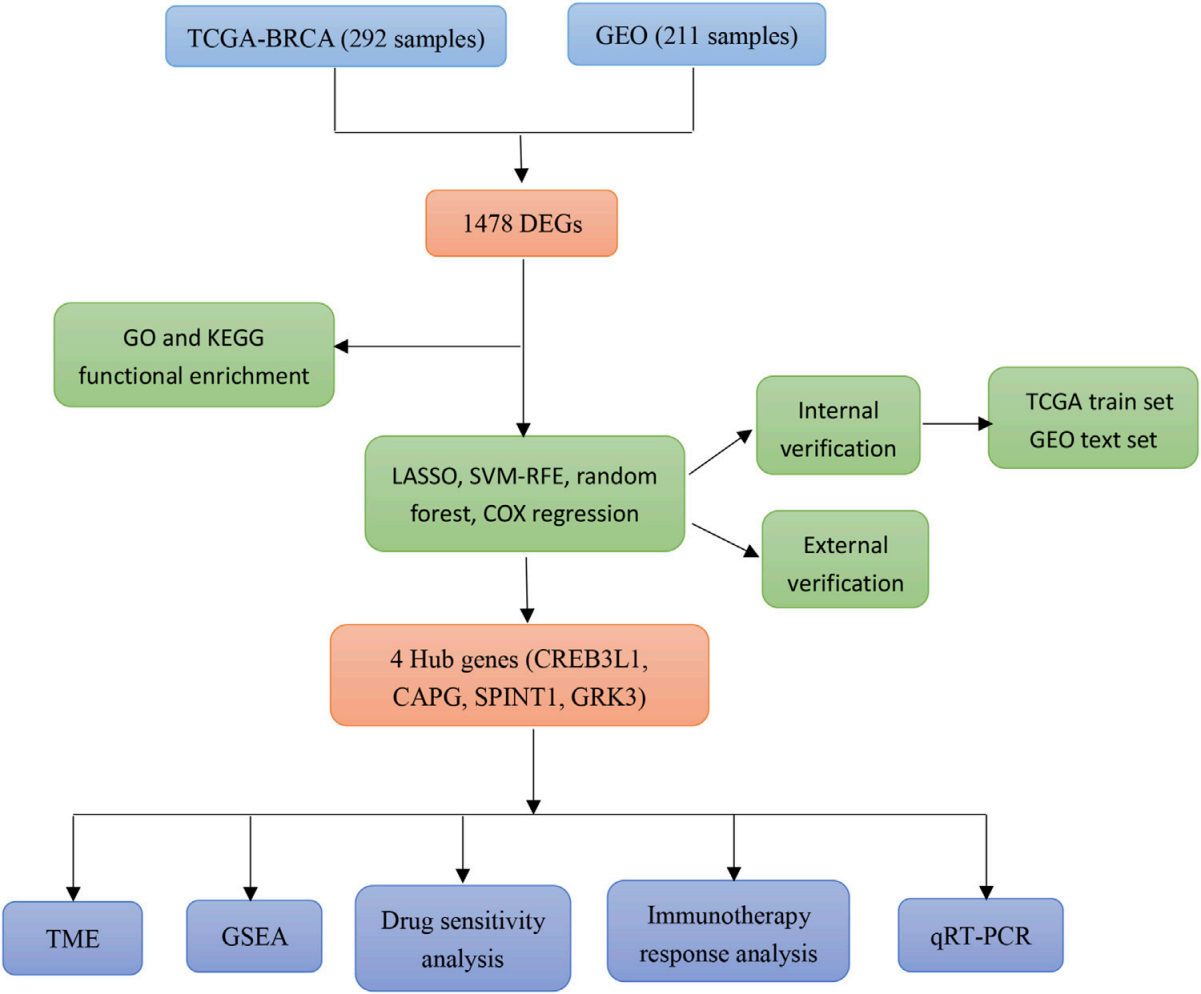
Flow chart of the present study.

In this study, a mitochondrial function-associated PCD-related risk model was constructed to predict the efficacy and prognosis of treatment intervention in early breast cancer. The flow chart of this study was shown in (Figure 1). This study not only expands the understanding of the invasiveness of early breast cancer, but also helps to formulate more personalized and precise treatment strategies for early breast cancer.

## 2 Materials and methods

### 2.1 Sources and preprocessing for data

The log2 (FPKM+1) expression data and clinical information of BRCA were acquired from TCGA database. The preprocessing of data is as follows: (a) Samples containing Stage I information of early breast cancer samples were retained, and other samples such as blanks were removed; (b) Samples lacking survival time or with zero survival time were eliminated from the analysis, ensuring that only TCGA patient samples with available prognostic information were included; (c) Samples with missing values and unexpressed genes exceeding 50% of the total sequencing number were excluded; (d) Genes not expressed in more than 50% of the samples and genes were removed; (e) All expression values were logarithmized using log2 (X + 1). Finally, total 179 early breast cancer and 113 normal samples were included. Besides, we also downloaded the datasets GSE42568 and GSE58812 from the GEO database as the validation datasets. After we removed samples without survival time or survival time = 0, total 104 and 107 early breast cancer samples were selected, respectively.

### 2.2 Identification of mitochondrial function-associated PCD related genes

Total 19 PCD patterns and key regulatory genes were collected through literature search (Chen et al., 2023; Hu et al., 2023), and after removing duplicates, total 1583 PCD-related genes were obtained. Besides, 1,136 mitochondrial function-related genes were obtained from MitoCarta 3.0 database (Rath et al., 2021).

### 2.3 Identification of DEGs

Screening of DEGs between early breast cancer and normal samples was conducted utilizing the “limma” package (version 3.34.7) (Liu et al., 2021). The Benjamin and Hochberg method was utilized for multiple test correction, and the corrected *p*-value (adj.*p*-value) was obtained. DEGs meeting the criteria of FDR <0.05 and |log2FC| ≥ 1 were obtained. In addition, “clusterProfiler” package (version 4.0.5) (Yu et al., 2012) was employed to conduct the enrichment analysis on the DEGs with the threshold of adj. *p*-value <0.05.

### 2.4 Identifying mitochondrial function-associated PCD related genes in breast cancer

The crosstalk genes in DEGs, PCD-related genes, and mitochondrial function-related genes were obtained, and “VennDiagram” package (version 1.7.1) was used to visualize. In addition, Pearson correlation analysis was performed on the RNA seq data of TCGA early breast cancer samples to determine the genes with threshold of correlation coefficient (|R|) > 0.6 and *p* < 0.001. Then, the STRING database (version 11.0) was employed to construct the protein-protein interaction (PPI) network of the crosstalk genes.

### 2.5 Establishment and validation of a prognostic risk model

To identify genes associated with prognosis, univariate Cox regression analysis was conducted on the crosstalk genes by the “survival” package (Rizvi et al., 2019) with the cutoff value of *p* < 0.05. Then, three machine learning algorithms were utilized to screen diagnostic genes, including LASSO logistic regression model, SVM-RFE model and random forest (RF) model, with “glmnet” package (Jiang et al., 2019), “e1071” (Functions and Wien, 2012), and “randomForest” (Liaw and Wiener, 2002) was utilized. The common genes obtained from three machine learning algorithms were acquired as the diagnostic genes, then the “survminer” package (version 0.4.9) was employed to conduct the multiple Cox logistic regression analysis, and the RiskScore was constructed utilizing the formula: RiskScore **=** β_1_X_1_ + β_2_X_2_ + … +β_n_X_n_ (where β indicates the regression coefficient, β_1_X_1_ + β_2_X_2_ + … +β_n_X_n_ indicates the linear combination of gene expression values X). The samples from TCGA training and GEO validation datasets were then broken into high- and low-risk groups based on the median risk score. Eventually, survival analysis was carried out utilizing the Kaplan-Meier curve method, and the ROC curve was drawn to assess the prognostic performance of RiskScore.

### 2.6 Correlation of clinical features and RiskScore

By integrating the clinical information data of early breast cancer, the distribution differences of RiskScore among different clinical information were analyzed, containing age, TNM, ER, HER2, PR, etc.

### 2.7 Nomogram development

By integrating the clinical information data of early breast cancer, the relationship between RiskScore and clinical features (age, stage, etc.) were analyzed, and univariate and multivariate COX regression analyses were conducted to identify independent prognostic factors with threshold of *p* < 0.05. Subsequently, a nomogram was developed the utilizing “rms” package (version 6.2-0) (Zhang et al., 2019).

### 2.8 Tumor microenvironment (TME)

“CIBERSORT” (Chen et al., 2018), “ssGSEA” (Xiao et al., 2020), and “MCP-counter” (Becht et al., 2016) algorithms were employed to calculate the fraction of immune cells. Moreover, the “ESTIMATE” package (Hu et al., 2019) was employed to obtain ESTIMATE, stromal, and immune scores. Moreover, the “ggcor” package (version 0.9.8.1) was used to calculate the correlation between RiskScore, diagnostic genes and immune cells.

### 2.9 GSEA

The GSEA analysis was utilized to analyze the significant hallmark gene sets (h.all. v7.4. symbols) and KEGG enrichment among the RiskScore group with the cutoff value of *p* < 0.05 and |NES| > 1.

### 2.10 Drug sensitivity analysis

The GDSC database was used to assess the sensitivity of each patient to chemotherapy drugs, and the IC50 was quantified with the “pRRophetic” package (Geeleher et al., 2014).

### 2.11 Immunotherapy response

Each patient to immune checkpoint treatment was assessed using TIDE database, which was represented as TIDE score. The immune cytolytic activity (CYT) score was calculated using the log-average expression values of GZMA and PRF1, and the Third level lymphoid structure (TLS) score of TLS feature genes (CCL2, CCL3, and CCL4, etc.) were calculated using “GSVA” algorithm. In addition, the gene expression data of immune checkpoint were extracted based on the expression data of early breast cancer, and Wilcoxon test was employed to compare the expression differences of immune checkpoint genes among different RiskScore groups.

### 2.12 qRT-PCR

Finally, the qRT-PCR was conducted to verify the 4 key genes, including CREB3L1, CAPG, SPINT1 and GRK3. The breast cancer cell line T47D was obtained from the American Type Culture Collection (ATCC), and cultured in RPMI 1640, supplemented with 10% fetal bovine serum at 37 °C. The primer sequences were listed in Table 1. GAPDH was used as an internal reference.

**TABLE 1:** The primer sequences.

Gene	Sequences (5′-3′)
CREB3L1 (F)	GGAGAATGCCAACAGGAC
CREB3L1 (R)	ACC​AGA​ACA​AAG​CAC​AAG​G
CAPG (F)	CGA​ACA​CTC​AGG​TGG​AGA​TT
CAPG (R)	TCC​AGT​CCT​TGA​AAA​ATT​GC
SPINT1 (F)	CTG​GGC​AGG​CAT​AGA​CTT​GA
SPINT1 (R)	TCT​GGG​TGG​TCT​GAG​CTA​GT
GRK3 (F)	GTC​ATC​TCT​GAA​CGC​TGG​CA
GRK3 (R)	GGC​CTC​CTT​GAA​GGT​TTC​GA

### 2.13 Cell apoptosis analysis

T47D cells in logarithmic growth phase were used for transfection. Lipo6000™ reagent was combined with pcDNA3.1-vector, pcDNA3.1-CREB3L1; si-NC and si-SPINT1 were mixed evenly and added into 6-well plates (100 μL per well) as vector group, pcDNA3.1-CREB3L1 group, si-NC group and si-SPINT1NC group, respectively. The cells of each group after transfection for 48 h were collected, rinsed twice with precooled PBS, and resuspended in buffer to adjust the cell concentration to 1 × 10^6^ cells/mL. A total of 100 μL was added to a 5 mL culture tube, and 5 μL of Annexin V-FITC and PI were added, respectively. After incubation at 37°C for 15 min, the apoptosis rate of each group was detected.

## 3 Results

### 3.1 Identifying mitochondrial function-associated PCD related genes in breast cancer

**FIGURE 2:**
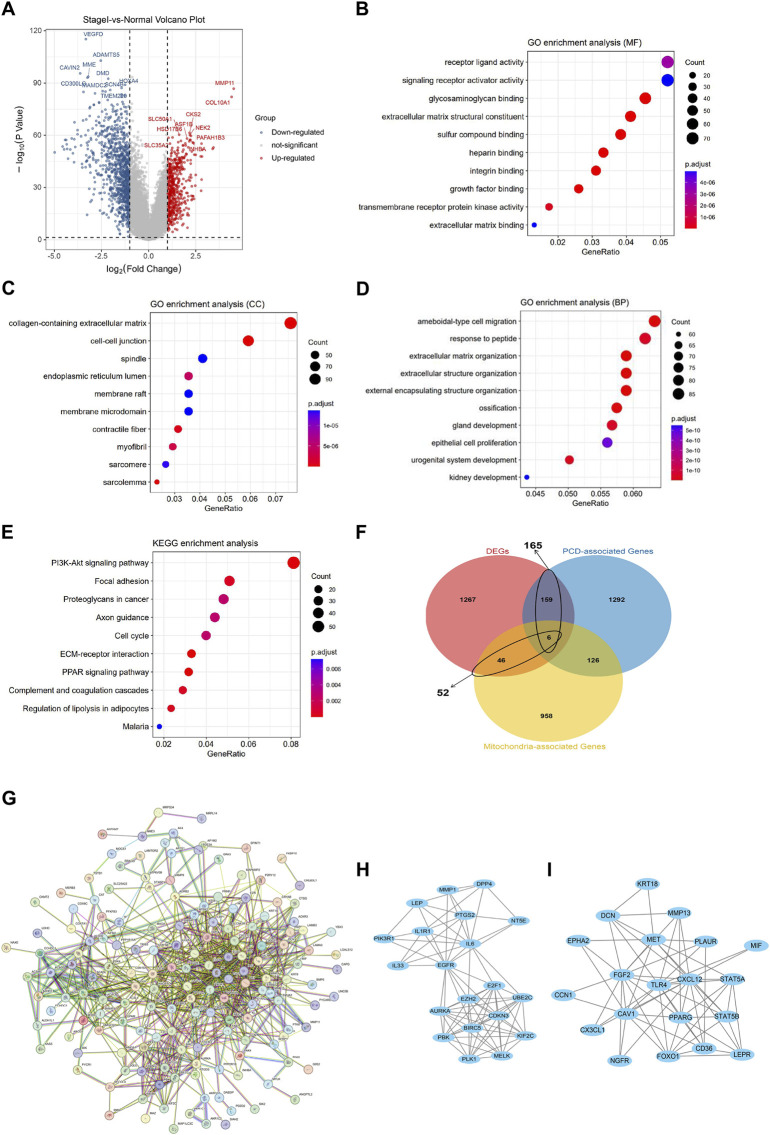
Identifying mitochondrial function-associated programmed cell death (PCD) related genes in breast cancer. **(A)** Volcano plot of differentially expressed genes (DEGs) between normal and breast cancer groups. The enriched GO-BP **(B)**, GO-CC **(C)**, and GO-MF **(D)**. **(E)** KEGG enrichment analysis. **(F)** Venn diagram of mitochondrial function-associated PCD related genes in breast cancer. **(G–I)** Protein-Protein Interaction (PPI) network of crosstalk genes.

A total of 1,478 DEGs were screened between normal and early breast cancer groups, containing 534 upregulated and 944 downregulated DEGs (Figure 2A). These 1,478 DEGs were involved in GO terms of ameboidal-type cell migration, glycosaminoglycan binding, and collagen-containing extracellular matrix (Figures 2B–D), and the involved KEGG pathways included PI3K-Akt signaling pathway, focal adhesion, and ECM-receptor interaction (Figure 2E). As shown in Figure 2F, total 178 crosstalk genes were obtained as mitochondrial function-associated PCD related gene ***s***, and the PPI network of the crosstalk genes was constructed (Figure 2G). In addition, we identified network markers with high median values and performed a relative permutation test (Figures 2H, I).

### 3.2 Establishment and validation of a prognostic risk model

**FIGURE 3:**
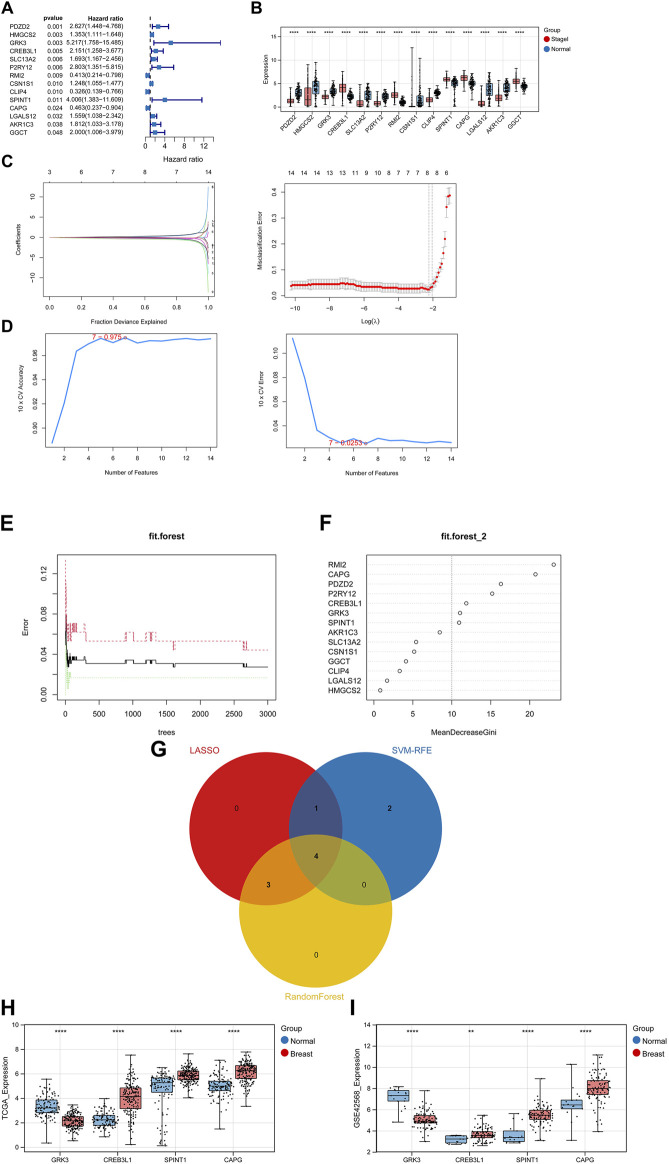
Identifying diagnostic genes. **(A)** 14 prognosis related genes obtained. **(B)** Expression of prognosis related crosstalk genes in normal and breast cancer groups. Feature genes were obtained using Least absolute shrinkage and selection operator (LASSO) logistic regression model **(C)**, SVM-RFE model **(D)**, and random forest model **(E,F)**. **(G)** Venn diagram of diagnostic genes. Expression of diagnostic genes in normal and breast cancer groups in TCGA training **(H)** and GSE42568 validation datasets **(I)**. **, *p* < 0.01, ***, *p* < 0.001, and ****, *p* < 0.0001.

**FIGURE 4:**
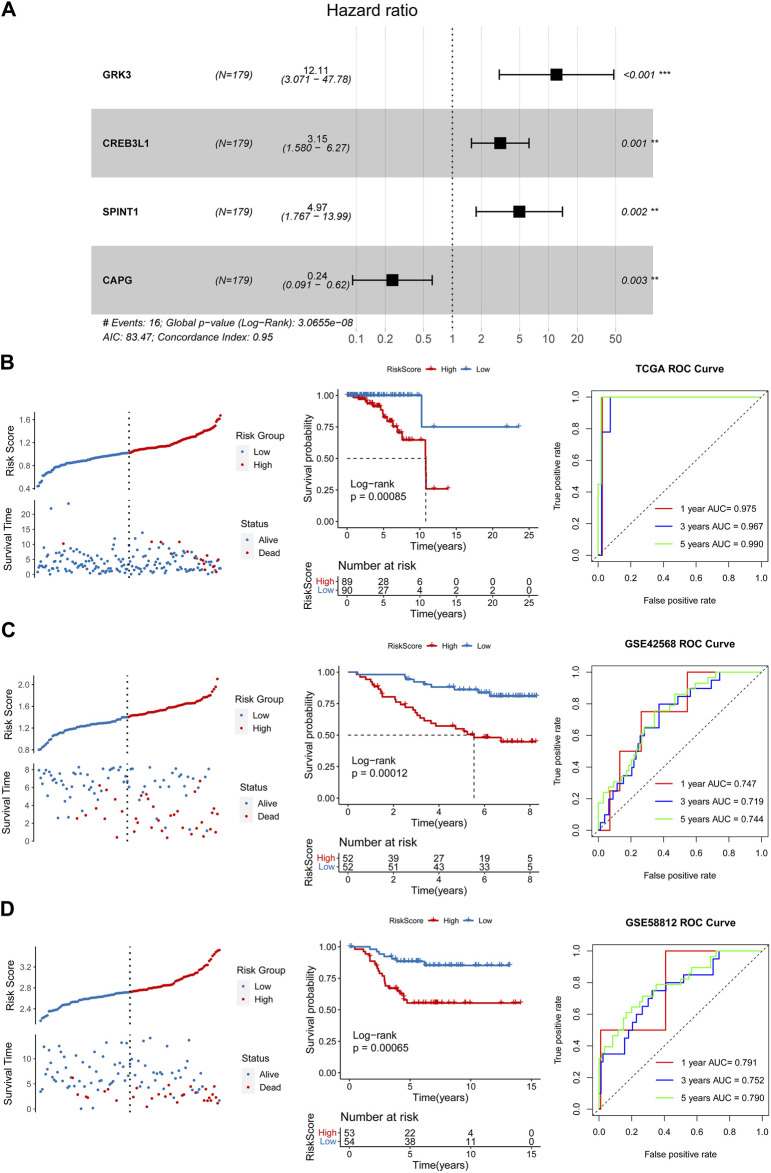
Construction and validation of a prognostic risk model. **(A)** Multiple Cox logistic regression analysis. Survival status, risk scores, prognosis, receiver operating characteristic (ROC) curves in the TCGA training **(B)**, GSE42568 **(C)** and GSE5881 **(D)** validation datasets.

After performing univariate Cox regression analysis, total 14 prognosis related crosstalk genes were obtained (Figure 3A). Also, the expression of these 14 prognosis related crosstalk genes in normal and breast cancer groups was illustrated in Figure 3B. Then, the feature genes were obtained using LASSO logistic regression model (Figure 3C), SVM-RFE model (Figure 3D), and random forest model (Figures 3E, F), respectively. Thus, total 4 common genes obtained from three machine learning algorithms were acquired as the diagnostic genes (Figure 3G), including CREB3L1, CAPG, SPINT1 and GRK3. Moreover, these 4 diagnostic genes showed good diagnostic ability in TCGA training (Figure 3H) and GSE42568 validation datasets (Figure 3I). After multiple Cox logistic regression analysis (Figure 4A), the RiskScore was built utilizing the formula: RiskScore = GRK3 * 0.2494 + CREB3L1 * 0.1147 +SPINT1 * 0.1604 + CAPG * (−0.1438). Besides, both the distribution of survival status, risk scores, prognosis, ROC curves in the TCGA and GEO validation datasets were displayed in Figures 4B–D, respectively. Patients classified into the high-risk group exhibited a noticeably poorer prognosis compared to those categorized into the low-risk group; the AUCs for OS at 1, 3, and 5 years were all above 0.7.

### 3.3 Correlation of clinical features and RiskScore

**FIGURE 5:**
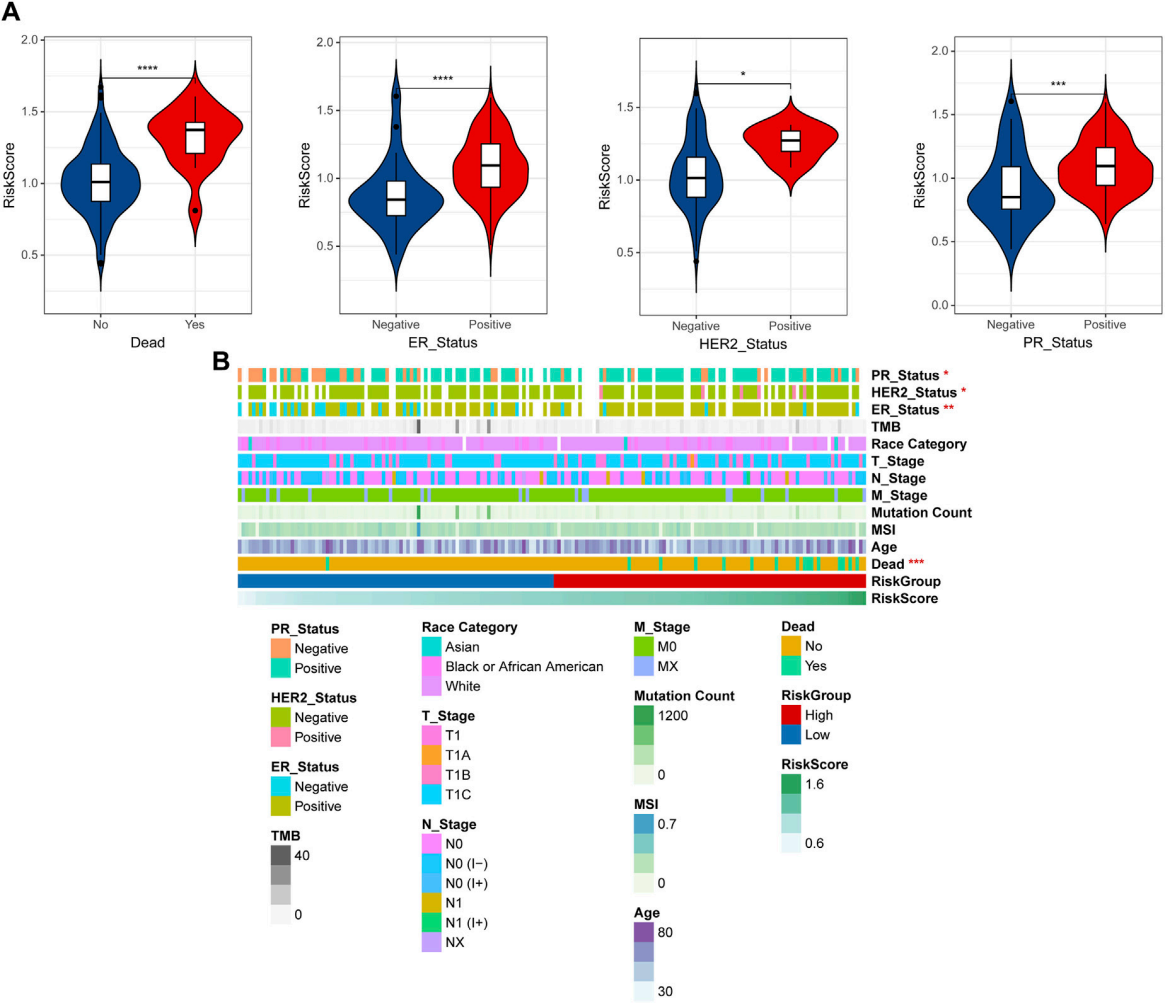
Correlation of clinical features and RiskScore. **(A)** The distribution differences of RiskScore among different clinical information. **(B)** Heatmap of clinical factors and RiskScore. *p < 0.05, **p < 0.01, ***p < 0.001, and ****p < 0.0001.

By integrating the clinical information data of early breast cancer, the distribution differences of RiskScore among different clinical information were analyzed, and the results shown that RiskScore showed significant differences among dead, ER, HER2, and PR (Figure 5A). To observe the relationship between clinical factors and RiskScore, the heatmap was shown in Figure 5B.

### 3.4 Nomogram

**FIGURE 6:**
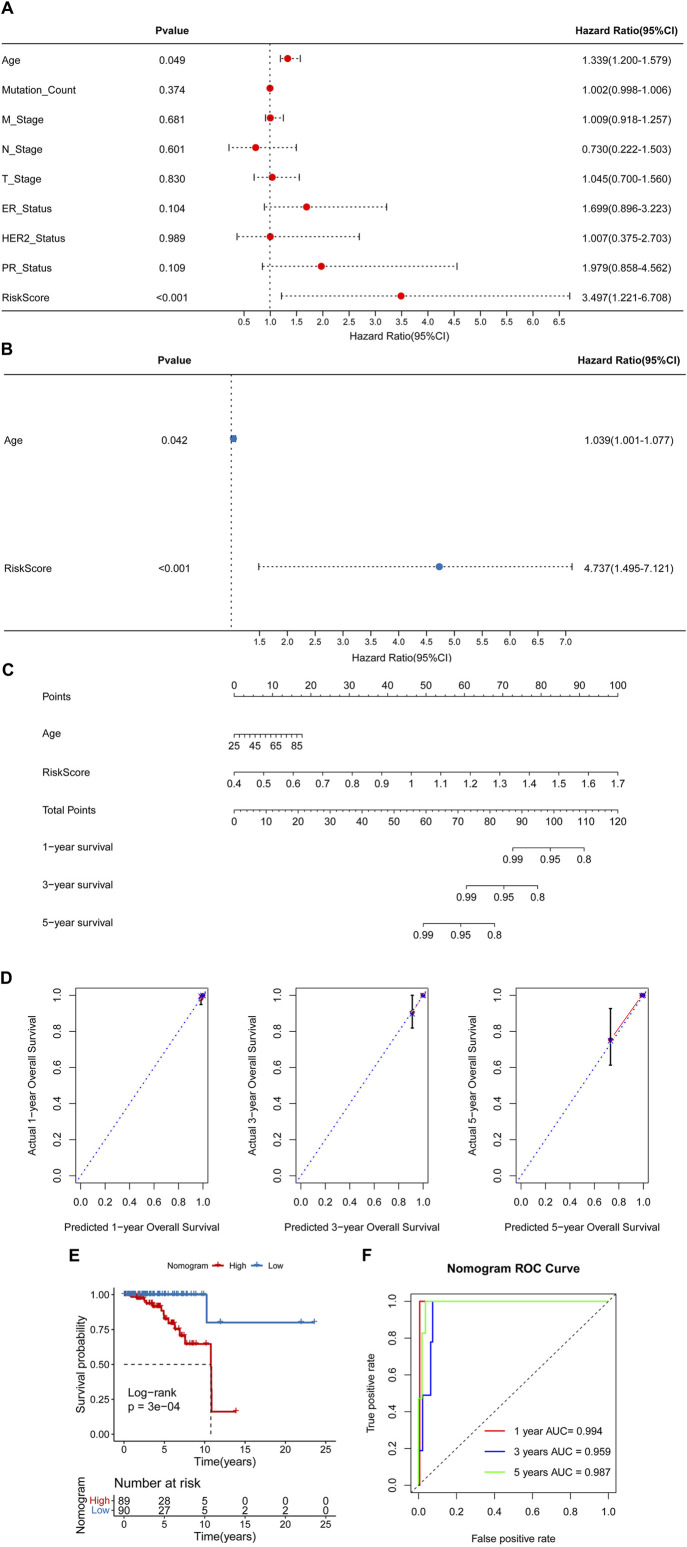
Construction of a nomogram. Univariate **(A)** and multivariate **(B)** Cox regression analyses. **(C)** A nomogram for predicting 1-, 3-, and 5-year overall survival (OS). **(D)** Calibration curves for predicting 1-, 3-, and 5-year OS. **(E)** Kaplan-Meier analysis of the nomogram. **(F)** The Area Under Curves (AUCs) for predicting 1-, 3-, and 5-year OS.

Univariate and multivariate Cox regression analyses were conducted to identify independent prognostic factors (Figures 6A, B), and age and RiskScore were identified as independent prognostic factors. A nomogram was then constructed using these factors (Figure 6C). The nomogram demonstrated accurate prediction of mortality (Figure 6D) and a significant association with patient prognosis (Figure 6E). The nomogram’s ROC revealed that the AUCs at 1, 3, and 5 years were 0.994, 0.959, and 0.987, separately (Figure 6F).

### 3.5 TME

**FIGURE 7:**
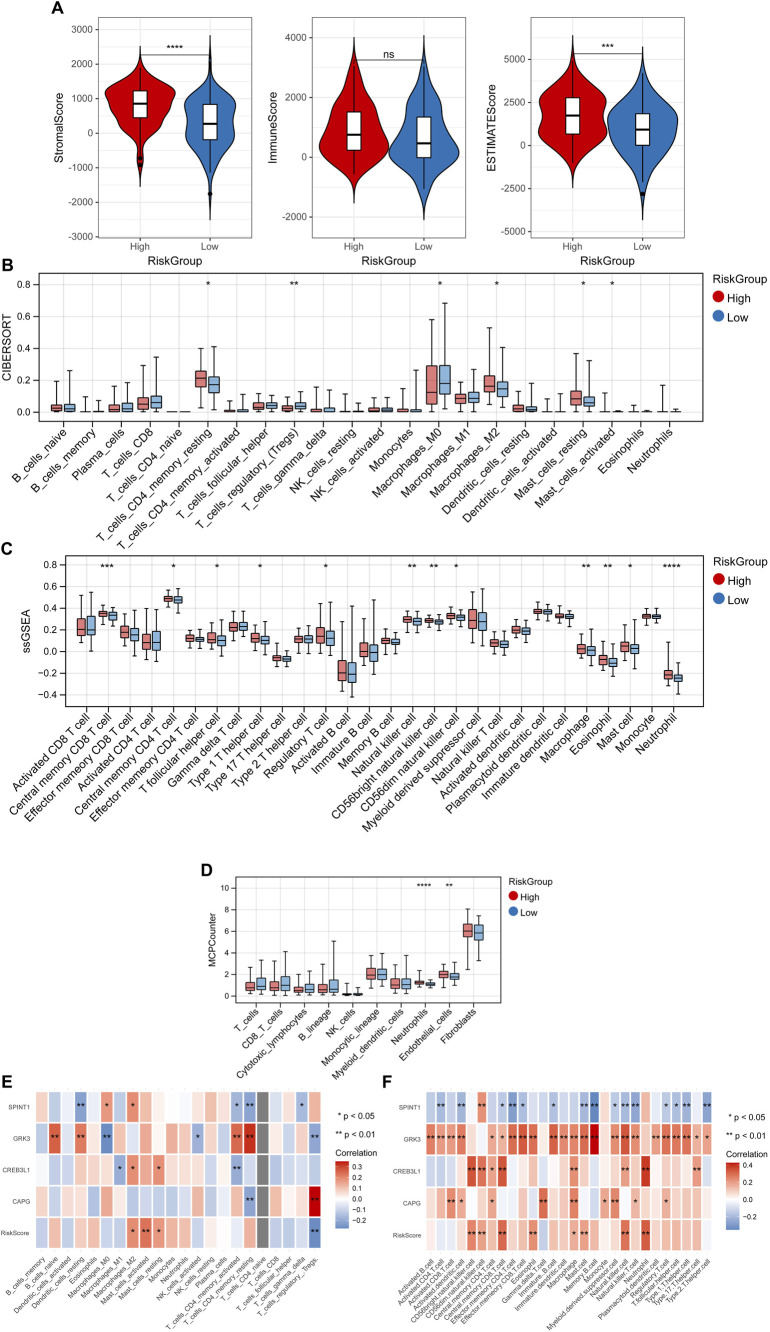
Tumor microenvironment. **(A)** Differences of StromalScore and ESTIMATEScore among high- and low-risk groups. Differences of fraction of immune cells between high- and low-risk groups utilizing “CIBERSORT” **(B)**, “ssGSEA” **(C)** and “MCP-counter” **(D)** algorithms. Correlation between the RiskScore, 4 key genes and immune cells in “CIBERSORT” **(E)**, and “ssGSEA” **(F)** algorithms. *p < 0.05, **p < 0.01, ***p < 0.001, and ns, no significant difference.

The StromalScore and ESTIMATEScore had significant differences among high- and low-risk groups (Figure 7A). The fraction of 6, 12 and 2 immune cells showed marked differences between high- and low-risk groups utilizing “CIBERSORT,” “ssGSEA” and “MCP-counter” algorithms, respectively (Figures 7B–D). Also, the correlation between the RiskScore, 4 key genes and immune cells were shown in Figures 7E, F.

### 3.6 GSEA, drug sensitivity analysis, and immunotherapy response analysis

**FIGURE 8:**
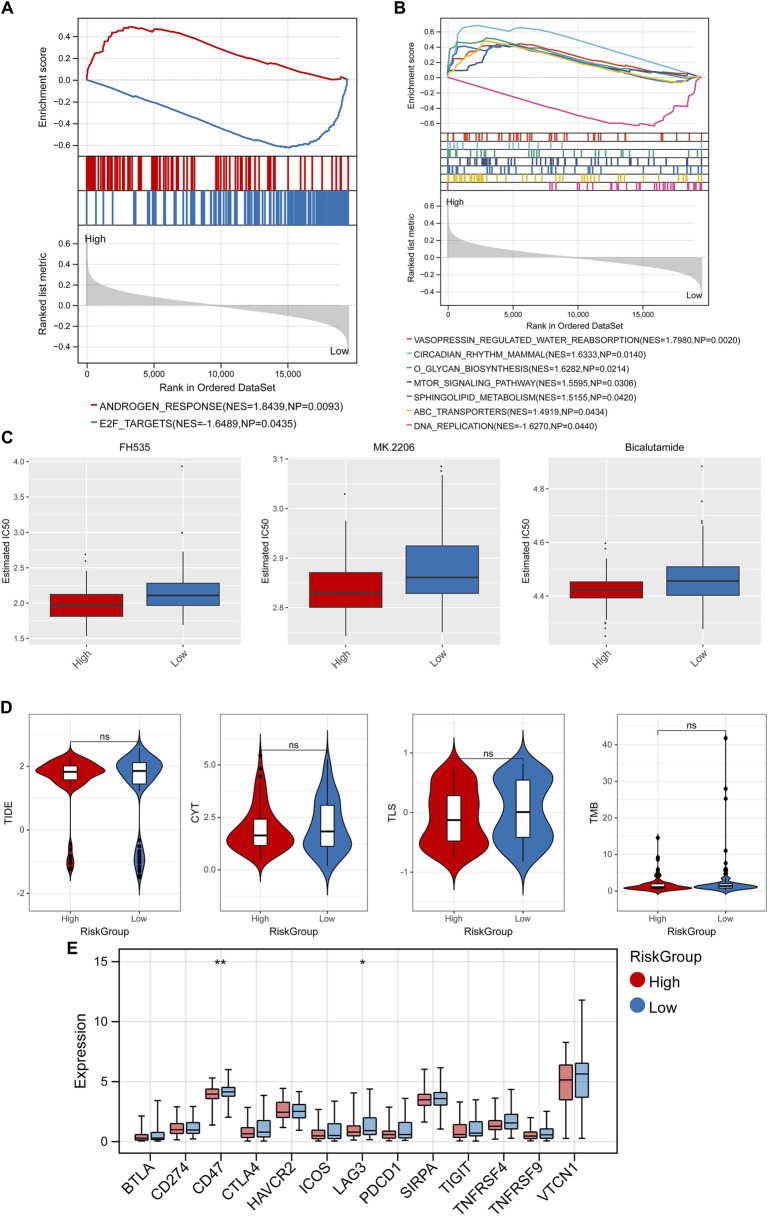
GSEA, drug sensitivity analysis, and immunotherapy response analysis. GSEA analysis found 2 significant hallmark gene sets **(A)** and 7 KEGG pathways **(B)**. **(C)** Top3 chemotherapeutics with significant differences between different RiskScore groups. **(D)** Difference of TIDE score, CYT, TLS, TMB in different RiskScore groups. **(E)** Difference of expression of immune checkpoint genes in different RiskScore groups. *p < 0.05, **p < 0.01, and ns, no significant difference.

GSEA analysis found 2 significant hallmark gene sets (Figure 8A) and 7 KEGG pathways (Figure 8B) among the RiskScore group with the cutoff value of *p* < 0.05 and |NES| > 1, including vasopressin regulated water reabsorption, circadian rhythm mammal, and o glycan biosynthesis. In addition, the differences in IC50 of 138 chemotherapeutics between different RiskScore groups were compared and the Top3 chemotherapeutics with significant differences were included FH535, MK.2206, and bicalutamide (Figure 8C). Besides, immunotherapy response analysis was carried out. No significant difference on TIDE score, CYT, TLS, TMB in different RiskScore groups (Figure 8D), while 2 immune checkpoint genes were lowly expressed in the high-risk group, including CD47 and LAG3 (Figure 8E).

### 3.7 Validation analysis

**FIGURE 9:**
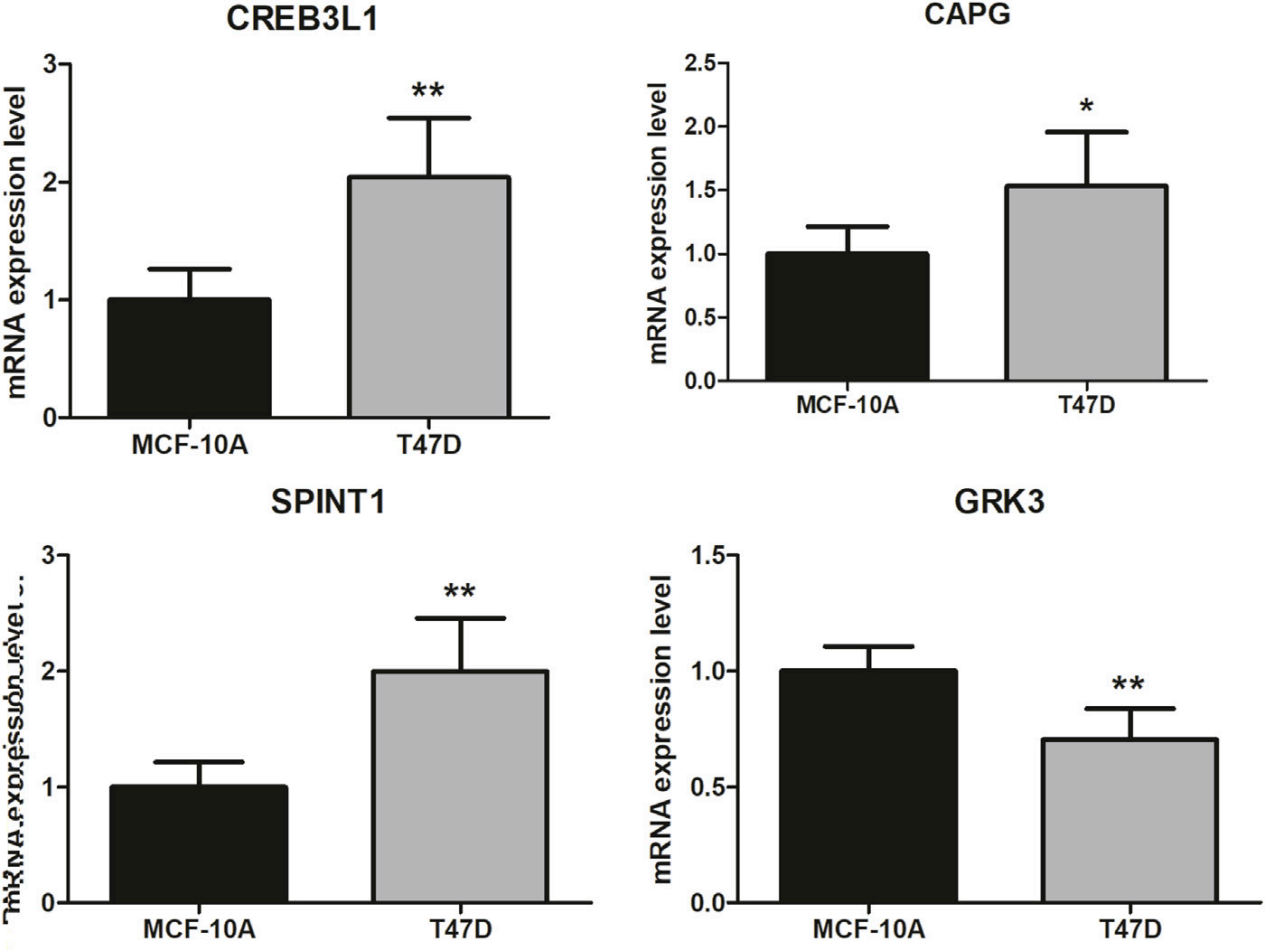
Validation analysis. The difference in clinical parameters between the two groups was analyzed using chi square test, and *p* < 0.05 was considered statistically significant. *p < 0.05, **p < 0.01.

Finally, the 4 key genes were verified using qRT-PCR, including CREB3L1, CAPG, SPINT1, and GRK3. As shown in Figure 9, the assays confirmed that CREB3L1, CAPG, and SPINT1 were significantly upregulated in breast cancer group, while GRK3 was significantly downregulated when compared to normal group, respectively.

### 3.8 Effect of CREB3L1 and SPINT1 on apoptosis of T47D cells

**FIGURE 10:**
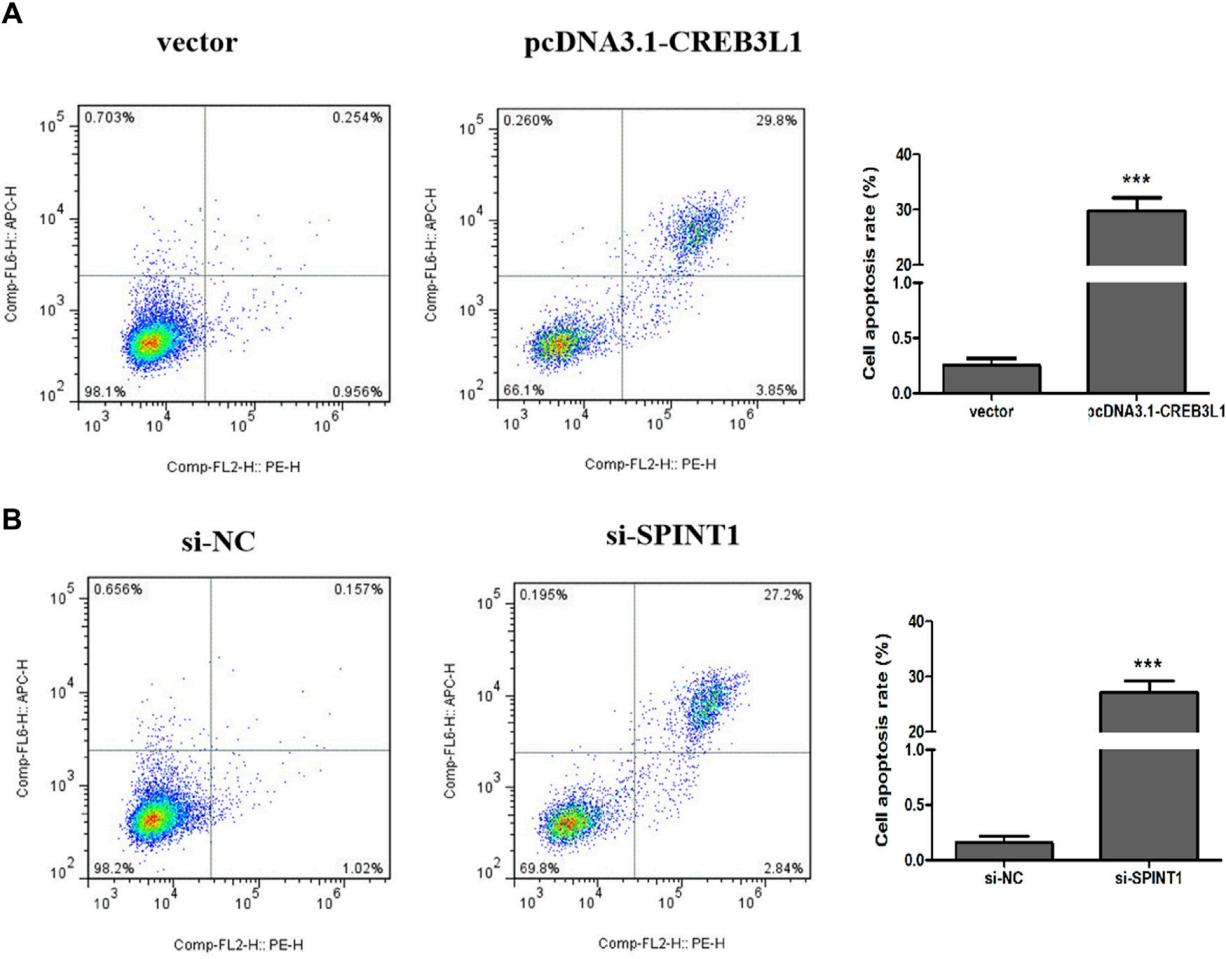
Effect of CREB3L1 and SPINT1 on apoptosis of T47D cells. ***p < 0.001.

Flow cytometry was used to detect the apoptosis of T47D cells 48 h after transfection. As shown in Figure 10, the total apoptosis rate of pcDNA3.1-CREB3L1 transfection group was significantly higher than that of vector control group (*p* < 0.001). Compared with the si-NC control group, the total apoptosis rate of the si-SPINT1 transfection group was significantly increased (*p* < 0.001).

## 4 Discussion

In this study, a total of 1,478 DEGs were screened between normal and early breast cancer groups, and these 1,478 DEGs were involved in PI3K-Akt signaling pathway, focal adhesion, and ECM-receptor interaction pathways. The PI3K/Akt pathway, which is crucial in various cellular processes, is abnormally activated in cancers and contributes to the occurrence and progression of tumors (He et al., 2021). Focal adhesion is essential in tumour invasiveness and metastasis (Shen et al., 2018). The extracellular matrix (ECM) is an essential component of the tumor microenvironment, and biological and mechanical alterations in the ECM have a profound impact on tumor invasion, metastasis, immune escape, and drug resistance (Gerarduzzi et al., 2020). In a primary tumor mass, the ECM is precisely regulated in a tumor-supporting manner, which consequently promotes tumor progression and affects the invasion of cancer cells (Mohan et al., 2020). Thus, we suspected that these DEGs might be involved in the early breast cancer development through PI3K-Akt signaling pathway, focal adhesion, and ECM-receptor interaction pathways.

It’s significant to determine gene signatures to predict prognosis or treatment responses based on specific gene sets or hallmarks in the field of oncology. In this study, in order to construct the prognostic risk model, three machine learning algorithms were utilized to screen diagnostic genes, including LASSO logistic regression model, SVM-RFE model and RF model, and total 4 common genes obtained from three machine learning algorithms were used to construct the prognostic risk model, containing CREB3L1, CAPG, SPINT1, and GRK3. The CREB3 family members are localized in the endoplasmic reticulum membrane and function as transcription factors after being cleaved by S1P and S2P proteases. In mammals, the CREB3 family comprises five members, which are crucial for protein secretion, survival, and lipid metabolism (García et al., 2017). It is believed that the CREB3L1 abnormal expression is the key driver of the malignant progression of numerous cancers (Rose et al., 2014; Feng et al., 2017; Puls et al., 2020). Pan et al. (2022) have found that CREB3L1 contributes to the tumor growth and metastasis of anaplastic thyroid carcinoma by altering the tumor microenvironment. Besides, endoplasmic reticulum stress (ERS) may reduce cell proliferation activity and promote cell apoptosis by mediating the expression of CREB3L1 in glioma (Yan et al., 2022). CAPG, also referred to as gCap39 or MCP, is a part of the gelsolin superfamily and plays a significant role in regulating actin assembly (Johnston et al., 1990). It’s reported that higher expression of CAPG has been observed in several metastatic cancers, indicating its involvement in cancer cell invasion and metastasis (Nag et al., 2013; Van Impe et al., 2013). Chi et al. (2019) also uncovered that CAPG enhances the resistance of breast cancer to paclitaxel by transactivating PIK3R1/P50. Huang et al. (2018) have revealed that CAPG promotes the metastasis of breast cancer by competing with PRMT5 to modulate the transcription of STC-1. In addition, CAPG could enhance the proliferation and invasion ability of diffuse large B-cell lymphoma (DLBCL) cells and inhibit cell apoptosis by activating the PI3K/AKT signaling pathway (Wang et al., 2022). SPINT1, also known as HAI1, is a type I transmembrane serine protease inhibitor that is commonly present on the surface of epithelial cells (Hoshiko et al., 2013). SPINT1 exerts significant effects on the development and progression of a variety of human malignant tumors, such as cell proliferation, invasion, migration, and metastasis (Shen et al., 2019). For example, Tian et al. have illustrated that exosome-mediated miR-221/222 targets SPINT1 to exacerbate tumor liver metastasis in colorectal cancer (Tian et al., 2021). Moreover, SPINT1-AS1 can promote the proliferation, migration and apoptosis of breast cancer cells by regulating miR-let-7a/b/i-5p, thus promoting the progress of breast cancer (Zhou et al., 2021). GRK3, also referred to as β-adrenergic receptor kinase 2, is a member of the GRK subfamily of kinases (Oliver et al., 2010). Previous studies showed that the GRK3 aberrant overexpression acts as a promoter mechanism in some kinds of tumors, including breast cancer and prostate cancer, especially in metastasis (Li et al., 2014; Billard et al., 2016). In colon cancer, downregulation of GRK3 expression reduces cell proliferation and migration, increases cell apoptosis, and impairs colon tumorigenesis in the xenograft model, suggesting that GRK3 promotes the malignant progression of colon cancer by mediating colon cancer cell proliferation (Jiang et al., 2017). The above reports fully demonstrate the reliability of our results, which were further validated by qRT-PCR. In addition, ROC curves in the TCGA and two GEO validation datasets were displayed, and the AUCs for OS at 1, 3, and 5 years were all above 0.7, which indicating that this prognostic risk model has good diagnostic performance for early breast cancer patients.

The TME plays a crucial role in the occurrence and development of tumors (Arneth, 2019). The TME contains multiple cell types, as well as many factors such as growth factors, signal transduction molecules, and the extracellular matrix, and these factors can alter the gene expression of tumor cells through multiple pathways, directly affecting the growth and metastasis of tumors (Xiao and Yu, 2021). In this study, three algorithms were employed to calculate the fraction of immune cells, including “CIBERSORT,” “ssGSEA” and “MCP-counter” algorithms, and the results shown that the fraction of some immune cells in high- and low-risk groups had significant difference, such as macrophage, eosinophil, mast cell, etc. Macrophages act as scavengers, modulating the immune response against pathogens and maintaining tissue homeostasis (Mehla and Singh, 2019). Tumor-associated macrophages are one of the most prevalent immune cells in the TME. In the early stages of tumor development, macrophages can either directly enhance antitumor responses through killing tumor cells or indirectly recruit and activate other immune cells (Lopez-Yrigoyen et al., 2021). Eosinophils are granulocytic leukocytes that reside in blood and tissues in the gastrointestinal, breast, and reproductive systems (O'Sullivan and Bochner, 2018). Normally, eosinophilia is not common in healthy individuals, however, it is associated with helminth infections, allergies, and some inflammatory conditions, as well as cancers (Davis and Rothenberg, 2014; Sakkal et al., 2016). Mast cells accumulate in the stroma around specific tumors and are involved in the inflammatory reaction at the edge of the tumor (Aller et al., 2019). The angiogenic cytokines secreted by mast cells not only have a direct effect on facilitating tumor vascularization, but also stimulate other inflammatory cells in the tumor microenvironment to release other angiogenic mediators (Cimpean et al., 2017). Therefore, we speculated that these immune cells might play essential roles in early breast cancer progression.

In addition, the differences in IC50 of 138 chemotherapeutics between different RiskScore groups were compared, and the Top3 chemotherapeutics with significant differences were included FH535, MK.2206, and bicalutamide. The results suggest that the RiskScore can effectively predict the sensitivity of breast cancer patients to common chemotherapy drugs. Li et al. (2023) constructed an EMT related lncRNA (ERL) signal that can accurately predict the prognosis of breast cancer patients. Through drug sensitivity analysis, it was found that the drug resistance of high-risk group to doxorubicin, gemcitabine, methodexate, palbiclib, and olaparib was higher than that of low-risk group, while the drug resistance of high-risk group to lapatinib was lower than that of low-risk group, suggesting that ERLs signals can effectively predict the sensitivity of breast cancer patients to commonly used chemotherapy drugs (Li et al., 2023). This study also performed the immunotherapy response analysis, and the results shown that 2 immune checkpoint genes were lowly expressed in the high-risk group, including CD47 and LAG3. CD47 is a transmembrane protein that is universally expressed on human cells, but is overexpressed on many types of tumor cells, which is an important tumor antigen (Hayat et al., 2020). Through precise and comprehensive reprogramming of the TME, the combination therapy containing CD47 and other immune checkpoint inhibitors be superior to that of monotherapy (Jiang et al., 2021). Upregulation of LAG3 is necessary to control excessive activation and prevent the occurrence of autoimmunity, and it’s reported that LAG3 could be used as a cancer immunotherapy target (Andrews et al., 2017). Thus, we suspected that two immune checkpoint genes might be used as early breast cancer immunotherapy target. Besides, the differences in IC50 of 138 chemotherapeutics between different RiskScore groups were compared and the Top3 chemotherapeutics with significant differences were included FH535, MK.2206, and bicalutamide. Wu et al. (2015) have found that FH535 inhibited metastasis and growth of pancreatic cancer cells. Wang et al. (2020) have revealed that Akt inhibitor MK-2206 reduces pancreatic cancer cell viability and increases the efficacy of gemcitabine. Therefore, FH535, MK.2206, and bicalutamide might be used for early breast cancer treatment.

**FIGURE 11:**
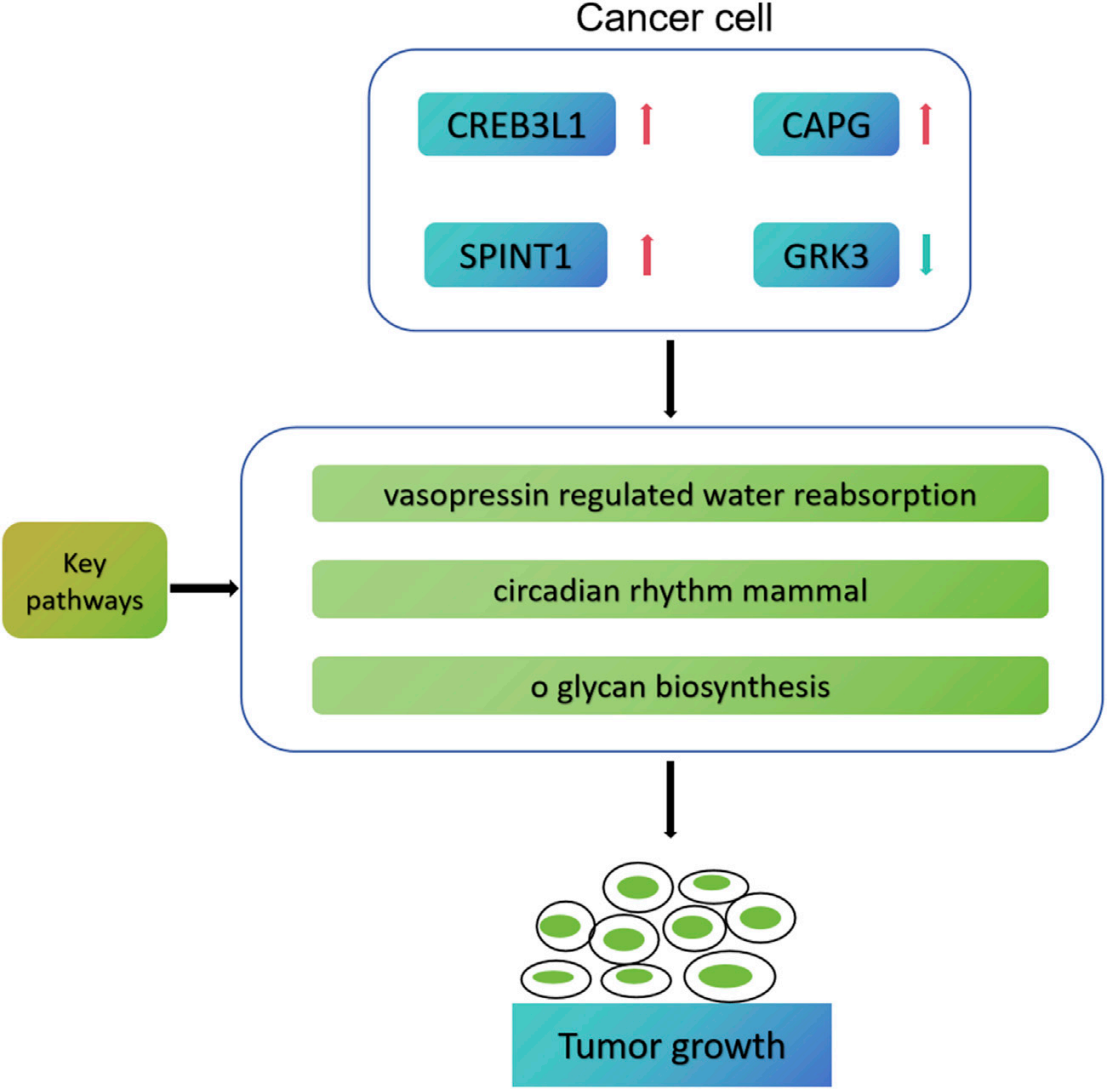
Possible molecular mechanism map of four key genes involved in early breast cancer progression.

Based on bioinformatics analysis, Ma et al. (2022) successfully screened 5 key genes related to the progression, prognosis and immunity of TNBC, namely, TOP2A, CCNA2, PCNA, MSH2 and CDK6. In addition, Wang et al. established a prognostic model for five genes (TNFRSF14, NFKBIA, DLG3, IRF2 and CYP27A1) based on the cell immune related gene module in TCGA-BRCA, which can effectively predict the prognosis and immune model of breast cancer patients (Wang et al., 2023). The previous research has similarities with the research methods of this study, but there are also differences. Ma et al. (2022) finally screened the pivotal genes related to TNBC prognosis and immunity, while we finally screened the cell death characteristic genes related to mitochondrial function in early breast cancer. Moreover, we speculate that CREB3L1, CAPG, SPINT1 and GRK3 may participate in the process of breast cancer through vasopressin regulated water reabsorption, circumferential rhithm mmal, and o glycan biosynthesis pathways (Figure 11). In addition, we also conducted drug sensitivity analysis and immunotherapy response analysis, laying the foundation for the next clinical validation.

This study also has some limitations. Firstly, the screened 4 key mitochondrial function-associated PCD-related genes, immune cells and chemotherapeutics should be further tested through experimental analyses. Secondly, whether the prognostic model and nomogram can be applied in clinic needs further study. Lastly, the function and mechanism of the 4 key mitochondrial function-associated PCD-related genes in early breast cancer need be further explored.

## 5 Conclusion

This study developed a new risk prognostic model for patients with early breast cancer based on 4 mitochondrial function-associated PCD-related genes. This risk prognostic model can precisely assess early breast cancer patients’ survival and offers potential biomarkers or treatment targets for early breast cancer patients. These findings may contribute to the development of therapeutic strategies targeting mitochondrial function-associated PCD for early breast cancer.

## Data Availability

The datasets presented in this study can be found in online repositories. The names of the repository/repositories and accession number(s) can be found below: http://www.ncbi.nlm.nih.gov/geo/query/acc.cgi?acc=GSE42568, GSE42568 https://www.ncbi.nlm.nih.gov/geo/query/acc.cgi?acc=GSE58812, GSE58812.

## References

[B1] Aller M. A. Arias A. Arias J. I. Arias J. (2019). Carcinogenesis: the cancer cell-mast cell connection. Inflamm. Res. official J. Eur. Histamine Res. Soc. 68 (2), 103–116. 10.1007/s00011-018-1201-4 30460391

[B2] Andrews L. P. Marciscano A. E. Drake C. G. Vignali D. A. (2017). LAG3 (CD223) as a cancer immunotherapy target. Immunol. Rev. 276 (1), 80–96. 10.1111/imr.12519 28258692 PMC5338468

[B3] Arneth B. (2019). Tumor microenvironment. Med. Kaunas. Lith. 56 (1), 15. 10.3390/medicina56010015 PMC7023392 31906017

[B4] Becht E. Giraldo N. A. Lacroix L. Buttard B. Elarouci N. Petitprez F. (2016). Estimating the population abundance of tissue-infiltrating immune and stromal cell populations using gene expression. Genome Biol. 17 (1), 218. 10.1186/s13059-016-1070-5 27765066 PMC5073889

[B5] Billard M. J. Fitzhugh D. J. Parker J. S. Brozowski J. M. McGinnis M. W. Timoshchenko R. G. (2016). G protein coupled receptor kinase 3 regulates breast cancer migration, invasion, and metastasis. PloS one 11 (4), e0152856. 10.1371/journal.pone.0152856 27049755 PMC4822790

[B6] Calvo S. E. Mootha V. K. (2010). The mitochondrial proteome and human disease. Annu. Rev. genomics Hum. Genet. 11, 25–44. 10.1146/annurev-genom-082509-141720 20690818 PMC4397899

[B7] Chen B. Khodadoust M. S. Liu C. L. Newman A. M. Alizadeh A. A. (2018). Profiling tumor infiltrating immune cells with CIBERSORT. Methods Mol. Biol. Clift. NJ 1711, 243–259. 10.1007/978-1-4939-7493-1_12 PMC5895181 29344893

[B8] Chen B. Xie K. Zhang J. Yang L. Zhou H. Zhang L. (2023b). Comprehensive analysis of mitochondrial dysfunction and necroptosis in intracranial aneurysms from the perspective of predictive, preventative, and personalized medicine. Apoptosis Int. J. Program. Cell death. 28 (9-10), 1452–1468. 10.1007/s10495-023-01865-x PMC10425526 37410216

[B9] Chen L. Zhou M. Li H. Liu D. Liao P. Zong Y. (2023a). Mitochondrial heterogeneity in diseases. Signal Transduct. Target. Ther. 8 (1), 311. 10.1038/s41392-023-01546-w 37607925 PMC10444818

[B10] Chi Y. Xue J. Huang S. Xiu B. Su Y. Wang W. (2019). CapG promotes resistance to paclitaxel in breast cancer through transactivation of PIK3R1/P50. Theranostics 9 (23), 6840–6855. 10.7150/thno.36338 31660072 PMC6815964

[B11] Cimpean A. M. Tamma R. Ruggieri S. Nico B. Toma A. Ribatti D. (2017). Mast cells in breast cancer angiogenesis. Crit. Rev. oncology/hematology. 115, 23–26. 10.1016/j.critrevonc.2017.04.009 28602166

[B12] Davis B. P. Rothenberg M. E. (2014). Eosinophils and cancer. Cancer Immunol. Res. 2 (1), 1–8. 10.1158/2326-6066.CIR-13-0196 24778159

[B13] Feng Y. X. Jin D. X. Sokol E. S. Reinhardt F. Miller D. H. Gupta P. B. (2017). Cancer-specific PERK signaling drives invasion and metastasis through CREB3L1. Nat. Commun. 8 (1), 1079. 10.1038/s41467-017-01052-y 29057869 PMC5651903

[B14] Functions T. M. Wien T. U. (2012). Package 'e1071'.

[B15] Galluzzi L. Joza N. Tasdemir E. Maiuri M. C. Hengartner M. Abrams J. M. (2008). No death without life: vital functions of apoptotic effectors. Cell death Differ. 15 (7), 1113–1123. 10.1038/cdd.2008.28 18309324 PMC2917777

[B16] García I. A. Torres Demichelis V. Viale D. L. Di Giusto P. Ezhova Y. Polishchuk R. S. (2017). CREB3L1-mediated functional and structural adaptation of the secretory pathway in hormone-stimulated thyroid cells. J. Cell Sci. 130 (24), 4155–4167. 10.1242/jcs.211102 29093023 PMC6518157

[B17] Geeleher P. Cox N. Huang R. S. (2014). pRRophetic: an R package for prediction of clinical chemotherapeutic response from tumor gene expression levels. PloS one 9 (9), e107468. 10.1371/journal.pone.0107468 25229481 PMC4167990

[B18] Gerarduzzi C. Hartmann U. Leask A. Drobetsky E. (2020). The matrix revolution: matricellular proteins and restructuring of the cancer microenvironment. Cancer Res. 80 (13), 2705–2717. 10.1158/0008-5472.CAN-18-2098 32193287

[B19] Guo Y. Guan T. Shafiq K. Yu Q. Jiao X. Na D. (2023). Mitochondrial dysfunction in aging. Ageing Res. Rev. 88, 101955. 10.1016/j.arr.2023.101955 37196864

[B20] Hayat S. M. G. Bianconi V. Pirro M. Jaafari M. R. Hatamipour M. Sahebkar A. (2020). CD47: role in the immune system and application to cancer therapy. Cell. Oncol. Dordr. 43 (1), 19–30. 10.1007/s13402-019-00469-5 31485984 PMC12990683

[B21] He Y. Sun M. M. Zhang G. G. Yang J. Chen K. S. Xu W. W. (2021). Targeting PI3K/Akt signal transduction for cancer therapy. Signal Transduct. Target. Ther. 6 (1), 425. 10.1038/s41392-021-00828-5 34916492 PMC8677728

[B22] Hoshiko S. Kawaguchi M. Fukushima T. Haruyama Y. Yorita K. Tanaka H. (2013). Hepatocyte growth factor activator inhibitor type 1 is a suppressor of intestinal tumorigenesis. Cancer Res. 73 (8), 2659–2670. 10.1158/0008-5472.CAN-12-3337 23447577

[B23] Hu D. Zhou M. Zhu X. (2019). Deciphering immune-associated genes to predict survival in clear cell renal cell cancer. BioMed Res. Int. 2019, 2506843. 10.1155/2019/2506843 31886185 PMC6925759

[B24] Hu G. Yao H. Wei Z. Li L. Yu Z. Li J. (2023). A bioinformatics approach to identify a disulfidptosis-related gene signature for prognostic implication in colon adenocarcinoma. Sci. Rep. 13 (1), 12403. 10.1038/s41598-023-39563-y 37524774 PMC10390519

[B25] Huang S. Chi Y. Qin Y. Wang Z. Xiu B. Su Y. (2018). CAPG enhances breast cancer metastasis by competing with PRMT5 to modulate STC-1 transcription. Theranostics 8 (9), 2549–2564. 10.7150/thno.22523 29721098 PMC5928908

[B26] Jiang B. Sun P. Tang J. Luo B. GLMNet: graph learning-matching networks for feature matching. 2019.

[B27] Jiang T. Yang C. Ma L. Wu Z. Ye L. Ma X. (2017). Overexpression of GRK3, promoting tumor proliferation, is predictive of poor prognosis in colon cancer. Dis. Markers 2017, 1202710. 10.1155/2017/1202710 29445249 PMC5763208

[B28] Jiang Z. Sun H. Yu J. Tian W. Song Y. (2021). Targeting CD47 for cancer immunotherapy. J. Hematol. Oncol. 14 (1), 180. 10.1186/s13045-021-01197-w 34717705 PMC8557524

[B29] Johnston P. A. Yu F. X. Reynolds G. A. Yin H. L. Moomaw C. R. Slaughter C. A. (1990). Purification and expression of gCap39. An intracellular and secreted Ca2(+)-dependent actin-binding protein enriched in mononuclear phagocytes. J. Biol. Chem. 265 (29), 17946–17952. 10.1016/s0021-9258(18)38255-3 2211671

[B30] Kamradt M. L. Makarewich C. A. (2023). Mitochondrial microproteins: critical regulators of protein import, energy production, stress response pathways, and programmed cell death. Am. J. physiology Cell physiology 325 (4), C807–C816. 10.1152/ajpcell.00189.2023 PMC11540166 37642234

[B31] Kopecka J. Gazzano E. Castella B. Salaroglio I. C. Mungo E. Massaia M. (2020). Mitochondrial metabolism: inducer or therapeutic target in tumor immune-resistance? Seminars Cell and Dev. Biol. 98, 80–89. 10.1016/j.semcdb.2019.05.008 31100351

[B32] Lei S. Zheng R. Zhang S. Chen R. Wang S. Sun K. (2021). Breast cancer incidence and mortality in women in China: temporal trends and projections to 2030. Cancer Biol. Med. 18 (3), 900–909. 10.20892/j.issn.2095-3941.2020.0523 34002584 PMC8330522

[B33] Li C. Zheng L. Xu G. Yuan Q. Di Z. Yang Y. (2023). Exploration of epithelial-mesenchymal transition-related lncRNA signature and drug sensitivity in breast cancer. Front. Endocrinol. (Lausanne) 14, 1154741. 10.3389/fendo.2023.1154741 37538794 PMC10396438

[B34] Li W. Ai N. Wang S. Bhattacharya N. Vrbanac V. Collins M. (2014). GRK3 is essential for metastatic cells and promotes prostate tumor progression. Proc. Natl. Acad. Sci. U. S. A. 111 (4), 1521–1526. 10.1073/pnas.1320638111 24434559 PMC3910602

[B35] Liaw A. Wiener MJRN. Classification and regression by randomForest. 2002; 23(23).

[B36] Liu S. Wang Z. Zhu R. Wang F. Cheng Y. Liu Y. (2021). Three differential expression analysis methods for RNA sequencing: limma, EdgeR, DESeq2. J. Vis. Exp. JoVE 175. 10.3791/62528 34605806

[B37] Lopez-Yrigoyen M. Cassetta L. Pollard J. W. (2021). Macrophage targeting in cancer. Ann. N. Y. Acad. Sci. 1499 (1), 18–41. 10.1111/nyas.14377 32445205

[B38] Ma J. Chen C. Liu S. Ji J. Wu D. Huang P. (2022). Correction to: identification of a five genes prognosis signature for triple-negative breast cancer using multi-omics methods and bioinformatics analysis. Cancer Gene Ther. 29 (11), 1803. 10.1038/s41417-022-00498-7 35778554

[B39] Mehla K. Singh P. K. (2019). Metabolic regulation of macrophage polarization in cancer. Trends cancer 5 (12), 822–834. 10.1016/j.trecan.2019.10.007 31813459 PMC7187927

[B40] Mohan V. Das A. Sagi I. (2020). Emerging roles of ECM remodeling processes in cancer. Seminars cancer Biol. 62, 192–200. 10.1016/j.semcancer.2019.09.004 31518697

[B41] Nag S. Larsson M. Robinson R. C. Burtnick L. D. (2013). Gelsolin: the tail of a molecular gymnast. Cytoskelet. Hob. NJ 70 (7), 360–384. 10.1002/cm.21117 23749648

[B42] Nguyen T. T. Wei S. Nguyen T. H. Jo Y. Zhang Y. Park W. (2023). Mitochondria-associated programmed cell death as a therapeutic target for age-related disease. Exp. Mol. Med. 55 (8), 1595–1619. 10.1038/s12276-023-01046-5 37612409 PMC10474116

[B43] Nolan E. Lindeman G. J. Visvader J. E. (2023). Deciphering breast cancer: from biology to the clinic. Cell 186 (8), 1708–1728. 10.1016/j.cell.2023.01.040 36931265

[B44] Nunnari J. Suomalainen A. (2012). Mitochondria: in sickness and in health. Cell 148 (6), 1145–1159. 10.1016/j.cell.2012.02.035 22424226 PMC5381524

[B45] Oliver E. Rovira E. Montó F. Valldecabres C. Julve R. Muedra V. (2010). beta-Adrenoceptor and GRK3 expression in human lymphocytes is related to blood pressure and urinary albumin excretion. J. Hypertens. 28 (6), 1281–1289. 10.1097/HJH.0b013e3283383564 20216086

[B46] O'Sullivan J. A. Bochner B. S. (2018). Eosinophils and eosinophil-associated diseases: an update. J. allergy Clin. Immunol. 141 (2), 505–517. 10.1016/j.jaci.2017.09.022 29045815 PMC5803328

[B47] Pan Z. Xu T. Bao L. Hu X. Jin T. Chen J. (2022). CREB3L1 promotes tumor growth and metastasis of anaplastic thyroid carcinoma by remodeling the tumor microenvironment. Mol. cancer 21 (1), 190. 10.1186/s12943-022-01658-x 36192735 PMC9531463

[B48] Puls F. Agaimy A. Flucke U. Mentzel T. Sumathi V. P. Ploegmakers M. (2020). Recurrent fusions between YAP1 and KMT2A in morphologically distinct neoplasms within the spectrum of low-grade fibromyxoid sarcoma and sclerosing epithelioid fibrosarcoma. Am. J. Surg. pathology 44 (5), 594–606. 10.1097/PAS.0000000000001423 31913156

[B49] Qiu H. Cao S. Xu R. (2021). Cancer incidence, mortality, and burden in China: a time-trend analysis and comparison with the United States and United Kingdom based on the global epidemiological data released in 2020. Cancer Commun. Lond. Engl. 41 (10), 1037–1048. 10.1002/cac2.12197 PMC8504144 34288593

[B50] Rath S. Sharma R. Gupta R. Ast T. Chan C. Durham T. J. (2021). MitoCarta3.0: an updated mitochondrial proteome now with sub-organelle localization and pathway annotations. Nucleic acids Res. 49 (D1), D1541–D1547. 10.1093/nar/gkaa1011 33174596 PMC7778944

[B51] Rizvi A. A. Karaesmen E. Morgan M. Preus L. Wang J. Sovic M. (2019). gwasurvivr: an R package for genome-wide survival analysis. Bioinforma. Oxf. Engl. 35 (11), 1968–1970. 10.1093/bioinformatics/bty920 PMC7963072 30395168

[B52] Rose M. Schubert C. Dierichs L. Gaisa N. T. Heer M. Heidenreich A. (2014). OASIS/CREB3L1 is epigenetically silenced in human bladder cancer facilitating tumor cell spreading and migration *in vitro* . Epigenetics 9 (12), 1626–1640. 10.4161/15592294.2014.988052 25625847 PMC4623247

[B53] Roy M. Fowler A. M. Ulaner G. A. Mahajan A. (2023). Molecular classification of breast cancer. Pet. Clin. 18 (4), 441–458. 10.1016/j.cpet.2023.04.002 37268505

[B54] Saha T. Dash C. Jayabalan R. Khiste S. Kulkarni A. Kurmi K. (2022). Intercellular nanotubes mediate mitochondrial trafficking between cancer and immune cells. Nat. Nanotechnol. 17 (1), 98–106. 10.1038/s41565-021-01000-4 34795441 PMC10071558

[B55] Sakkal S. Miller S. Apostolopoulos V. Nurgali K. (2016). Eosinophils in cancer: favourable or unfavourable? Curr. Med. Chem. 23 (7), 650–666. 10.2174/0929867323666160119094313 26785997

[B56] Shen F. F. Pan Y. Yang H. J. Li J. K. Zhao F. Su J. F. (2019). Decreased expression of SPINT1-AS1 and SPINT1 mRNA might be independent unfavorable prognostic indicators in esophageal squamous cell carcinoma. OncoTargets Ther. 12, 4755–4763. 10.2147/OTT.S206448 PMC6591775 31417276

[B57] Shen J. Cao B. Wang Y. Ma C. Zeng Z. Liu L. (2018). Hippo component YAP promotes focal adhesion and tumour aggressiveness via transcriptionally activating THBS1/FAK signalling in breast cancer. J. Exp. Clin. cancer Res. CR 37 (1), 175. 10.1186/s13046-018-0850-z 30055645 PMC6064138

[B58] Sung H. Ferlay J. Siegel R. L. Laversanne M. Soerjomataram I. Jemal A. (2021). Global cancer statistics 2020: GLOBOCAN estimates of incidence and mortality worldwide for 36 cancers in 185 countries. CA a cancer J. Clin. 71 (3), 209–249. 10.3322/caac.21660 33538338

[B59] Tian F. Wang P. Lin D. Dai J. Liu Q. Guan Y. (2021). Exosome-delivered miR-221/222 exacerbates tumor liver metastasis by targeting SPINT1 in colorectal cancer. Cancer Sci. 112 (9), 3744–3755. 10.1111/cas.15028 34125460 PMC8409403

[B60] Van Impe K. Bethuyne J. Cool S. Impens F. Ruano-Gallego D. De Wever O. (2013). A nanobody targeting the F-actin capping protein CapG restrains breast cancer metastasis. Breast cancer Res. BCR 15 (6), R116. 10.1186/bcr3585 24330716 PMC3979033

[B61] Wang G. Liu H. An L. Hou S. Zhang Q. (2022). CAPG facilitates diffuse large B-cell lymphoma cell progression through PI3K/AKT signaling pathway. Hum. Immunol. 83 (12), 832–842. 10.1016/j.humimm.2022.10.001 36244872

[B62] Wang R. Zeng H. Xiao X. Zheng J. Ke N. Xie W. (2023). Identification of prognostic biomarkers of breast cancer based on the immune-related gene module. Autoimmunity 56 (1), 2244695. 10.1080/08916934.2023.2244695 37584152

[B63] Wang Z. Luo G. Qiu Z. (2020). Akt inhibitor MK-2206 reduces pancreatic cancer cell viability and increases the efficacy of gemcitabine. Oncol. Lett. 19 (3), 1999–2004. 10.3892/ol.2020.11300 32194695 PMC7039141

[B64] Wu M. Y. Liang R. R. Chen K. Shen M. Tian Y. L. Li D. M. (2015). FH535 inhibited metastasis and growth of pancreatic cancer cells. OncoTargets Ther. 8, 1651–1670. 10.2147/OTT.S82718 PMC4500609 26185454

[B65] Xiao B. Liu L. Li A. Xiang C. Wang P. Li H. (2020). Identification and verification of immune-related gene prognostic signature based on ssGSEA for osteosarcoma. Front. Oncol. 10, 607622. 10.3389/fonc.2020.607622 33384961 PMC7771722

[B66] Xiao Y. Yu D. (2021). Tumor microenvironment as a therapeutic target in cancer. Pharmacol. Ther. 221, 107753. 10.1016/j.pharmthera.2020.107753 33259885 PMC8084948

[B67] Yan Z. Hu Y. Zhang Y. Pu Q. Chu L. Liu J. (2022). Effects of endoplasmic reticulum stress-mediated CREB3L1 on apoptosis of glioma cells. Mol. Clin. Oncol. 16 (4), 83. 10.3892/mco.2022.2516 35251634 PMC8892456

[B68] Yu G. Wang L. G. Han Y. He Q. Y. (2012). clusterProfiler: an R package for comparing biological themes among gene clusters. Omics a J. Integr. Biol. 16 (5), 284–287. 10.1089/omi.2011.0118 PMC3339379 22455463

[B69] Zhang S. Tong Y. X. Zhang X. H. Xu X. S. Xiao A. T. (2019). A novel and validated nomogram to predict overall survival for gastric neuroendocrine neoplasms. J. Cancer 10 (24), 5944–5954. 10.7150/jca.35785 31762804 PMC6856574

[B70] Zhou T. Lin K. Nie J. Pan B. He B. Pan Y. (2021). LncRNA SPINT1-AS1 promotes breast cancer proliferation and metastasis by sponging let-7 a/b/i-5p. Pathol. Res. Pract. 217, 153268. 10.1016/j.prp.2020.153268 33246290

